# CT-defined sarcopenia as a predictor of early mortality following hip fracture: a retrospective cohort study

**DOI:** 10.1007/s00590-026-04981-x

**Published:** 2026-09-30

**Authors:** Vanya Sachdeva, Beatrice Walcot, Jessica Nightingale, Alan Norrish

**Affiliations:** 1 Academic Unit of Injury, Recovery and Inflammation Sciences, School of Medicine, University of Nottingham, Nottingham, UK; 2 Queens Medical Centre, Nottingham University Hospitals NHS Trust, Nottingham, UK

**Keywords:** Hip fracture, Sarcopenia, Computed tomography, Skeletal muscle area, Mortality

## Abstract

**Purpose:**

Sarcopenia is increasingly recognised as a marker of physiological vulnerability. Computed tomography permits objective quantification of skeletal muscle mass, yet the prognostic significance of CT-defined sarcopenia after hip fracture remains incompletely characterised. This study aimed to determine sarcopenia prevalence in patients with hip fractures and evaluate associations with early postoperative outcomes.

**Methods:**

This single-centre retrospective cohort study included patients with hip fracture during 2015 to 2025 who had abdominal computed tomography imaging, including the third lumbar vertebra (L3), within one year before fracture. Skeletal muscle area at L3 was quantified by manual segmentation on the Picture Archiving and Communication System (PACS) according to a predefined protocol. The cohort comprised 486 patients: 250 sarcopenic and 236 non-sarcopenic. Thirty-day and ninety-day mortality were the primary outcomes, analysed using adjusted logistic regression and 90-day time-to-event analysis. Length of hospital stay and discharge destination were secondary outcomes.

**Results:**

Sarcopenia was independently associated with increased mortality at 30 days (adjusted OR 3.88, 95% CI 1.83 to 8.22) and 90 days (adjusted OR 3.75, 95% CI 2.22 to 6.35). Cox regression similarly demonstrated increased 90-day mortality hazard (adjusted HR 3.19, 95% CI 2.01 to 5.06). No significant associations were observed between sarcopenia and length of hospital stay (median 13.27 vs. 13.25 days; *p* = 0.930) or discharge destination (χ^2^= 1.63, *p* = 0.202).

**Conclusion:**

CT-defined sarcopenia was independently associated with early mortality, supporting its potential role as an opportunistic marker of postoperative risk.

**Supplementary Information:**

The online version contains supplementary material available at https://doi.org/10.1007/s00590-026-04981-x.

## Introduction

Sarcopenia is a progressive skeletal muscle disorder characterised by reduced muscle strength, diminished muscle quantity or quality, and impaired physical performance [1]. Although initially described in relation to age-associated loss of muscle mass, the concept of sarcopenia has undergone significant conceptual reappraisal and is increasingly recognised as a marker of reduced physiological reserve [2]. This shift reflects a growing appreciation of skeletal muscle as more than an organ of locomotion; it is central to metabolic stability, functional independence, and recovery from physiological stress [3]. In older adults, loss of muscle reserve may therefore compromise resilience during acute illness, operative intervention, and postoperative catabolism [4].

This vulnerability is particularly relevant in hip fracture, where outcomes depend not only on surgical fixation, but also on the patient’s capacity to regain mobility after a major physiological insult [5]. Sarcopenia may thus contribute both to vulnerability before fracture through impaired strength and postural stability, and poorer outcomes after injury [6, 7]. Existing evidence has linked sarcopenia with adverse outcomes after trauma and fracture, although its prognostic significance in hip fracture populations remains incompletely defined [7].

Accurate assessment of muscle mass is central to evaluating this relationship. Computed tomography (CT) provides an objective means of quantifying skeletal muscle mass using routinely acquired imaging [8]. The third lumbar vertebral level (L3) is widely adopted in CT-based body composition analysis because skeletal muscle area at this site provides a validated surrogate for whole-body skeletal muscle mass [9]. As L3 is commonly captured on abdominal CT, this approach permits opportunistic assessment without additional imaging burden and supports comparison with established L3-based sarcopenia thresholds and previous prognostic studies [10]. This study aimed to determine the prevalence of CT-defined sarcopenia in patients admitted with hip fracture and to evaluate its association with 30-day and 90-day mortality.

## Methods

### Study design and participants

**Fig. 1:**
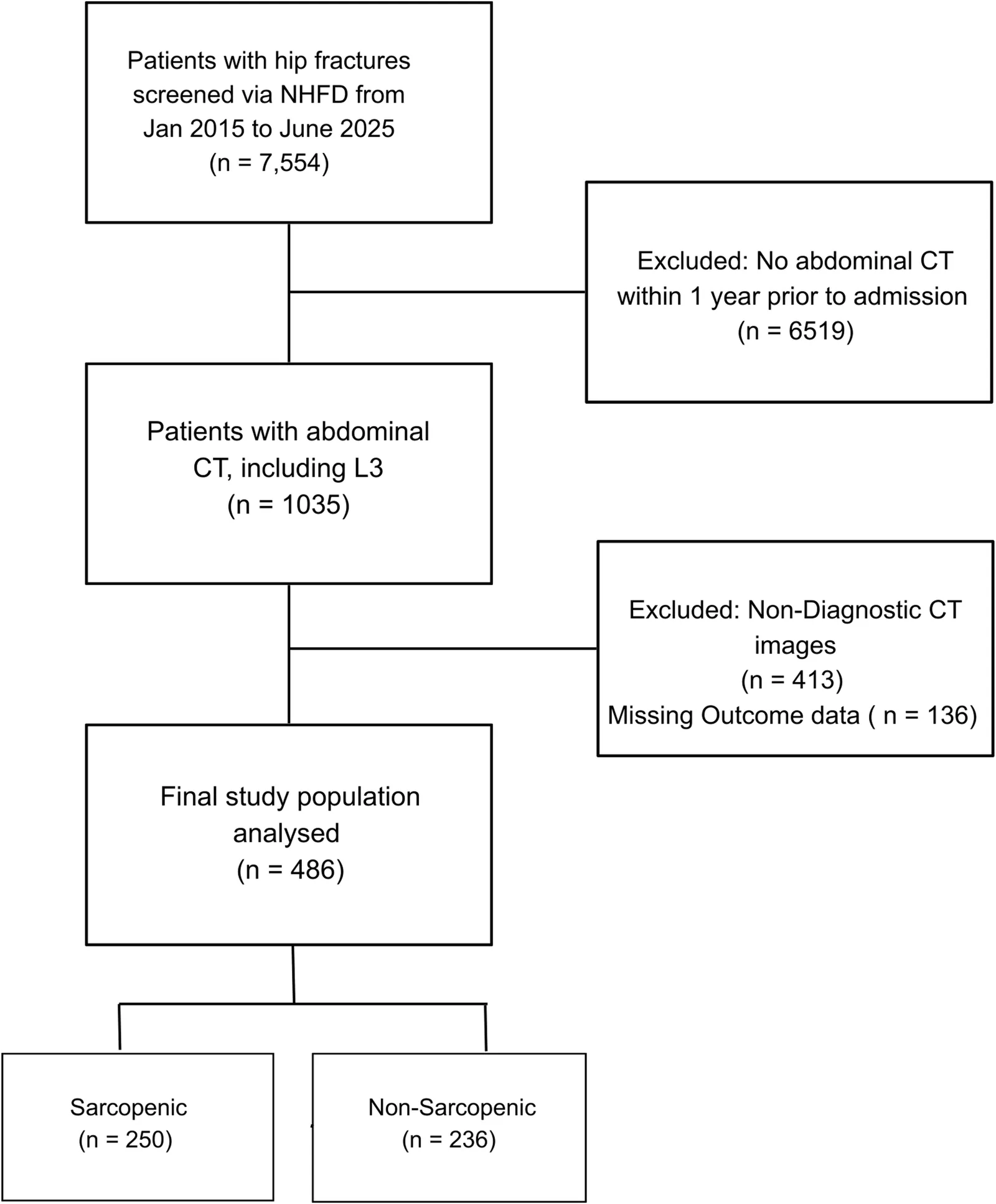
Patient inclusion and exclusion pathway for the final study cohort

A retrospective cohort study used routinely collected clinical and imaging data from patients admitted with hip fracture to a UK NHS Trust between January 2015 and June 2025. Patients were identified through a national hip fracture database, and abdominal computed tomography images were retrieved via the local Picture Archiving and Communication System (PACS). All data were anonymised before analysis. Patients were eligible if they had an abdominal computed tomography scan within one year before the index hip fracture admission that included the third lumbar vertebral level. Patients were excluded if no suitable scan was available, if image quality was non-diagnostic because of motion artefact or incomplete field of view, if vertebral level identification was unreliable, or if outcome data were missing. Of 7,554 screened admissions, 486 patients met the inclusion criteria and were included in the final analysis (Fig. 1).

### CT analysis and definition of sarcopenia

Skeletal muscle area at L3 was quantified by manual segmentation on PACS. The L3 level was identified by locating the sacrum and counting cranially from L5; slices showing rib-bearing vertebrae were excluded to avoid vertebral misclassification. The psoas, paraspinal muscles, and abdominal wall muscles were included in the measurement. Image analysis was performed by two trained assessors, each analysing a separate subset of scans according to a predefined protocol. Assessors were blinded to patient outcomes. Functional measures such as grip strength, gait speed or chair-stand testing were not routinely assessed or recorded as part of clinical practice for hip fracture patients at the study centre. Sarcopenia was therefore operationalised using CT-derived skeletal muscle area at L3 and defined using sex-specific cut-offs reported by Derstine et al.: < 92.2 cm^2^ for females and < 144.3 cm^2^ for males [11]. Height-normalised indices were not used because height data were frequently missing or unreliable in this hip fracture cohort.

### Outcomes and covariates

The primary outcomes were 30-day and 90-day mortality. Mortality data were obtained through linkage with the Personal Demographics Service, capturing both in-hospital and community deaths. Secondary variables included length of hospital stay and discharge destination. Additional covariates included pre-admission mobility, pre-admission residence, haemoglobin, malignancy, abbreviated mental test (AMT), 4 A’s Test for delirium screening (4AT), Charlson Comorbidity Index (CCI), and American Society of Anesthesiologists physical status grade (ASA). Non-mortality outcomes and covariates were obtained from hospital electronic records.

### Statistical analysis

Statistical analyses were performed using IBM SPSS Statistics for Windows, version 29 (IBM Corp., 2022). Continuous variables were assessed for normality using the Shapiro-Wilk test and compared using independent-samples t-tests or Mann-Whitney U tests, as appropriate. Categorical variables are presented as frequencies with percentages and were compared using chi-square tests. To assess potential selection bias, baseline characteristics were compared between the included CT cohort and patients without pre-fracture CT imaging for whom baseline data were available. Unadjusted logistic regression estimated crude associations between sarcopenia and 30-day and 90-day mortality, followed by multivariable logistic regression adjusted for age, sex and CCI. A parsimonious adjusted model was chosen to minimise overfitting and further case loss from missing covariate data. Model calibration was assessed using the Hosmer-Lemeshow goodness-of-fit test. Kaplan-Meier analysis compared 90-day survival between sarcopenic and non-sarcopenic patients using the log-rank test. Patients without a recorded death within 90 days, including those who died after 90 days, were censored at 90 days. Cox proportional hazards regression estimated unadjusted and adjusted hazard ratios for 90-day mortality, with the adjusted model including age, sex and CCI. The proportional hazards assumption was assessed using visual inspection of log-minus-log survival plots and was not found to be violated. Analyses used available-case data; missing data were not imputed. All p-values were two-sided, and *p* < 0.05 was considered statistically significant.

## Results

A total of 486 patients were included, of whom 250 (51.4%) were sarcopenic and 236 (48.6%) non-sarcopenic. Baseline characteristics are presented in Table 1. Age was similar between groups (81.1 ± 9.1 vs. 79.7 ± 8.8 years; *p* = 0.081). Sarcopenic patients had a higher proportion of males (54.8% vs. 19.5%; *p* < 0.001) and higher CCI (6.95 ± 2.49 vs. 6.43 ± 2.24; *p* = 0.017), although the effect size for comorbidity was small (Cohen’s d = 0.22). Other baseline variables were similar between groups.

To assess potential selection bias, baseline characteristics were compared between the included CT cohort and no-CT cohort with available baseline data (Supplementary Table 1). Compared with the no-CT cohort (*n* = 5,386), the included CT cohort (*n* = 486) was slightly younger (median 82 vs. 84 years), with higher CCI (7 vs. 5), male proportion (37.7% vs. 29.6%) and recorded malignancy prevalence (44.2% vs. 17.8%; all *p* < 0.001). ASA distribution also differed (*p* < 0.001), with more ASA 4–5 patients in the included CT cohort (28.6% vs. 19.4%). AMT score was slightly higher in the included CT cohort (9 vs. 8; *p* = 0.014).

Secondary clinical outcomes did not differ significantly by sarcopenia status. Acute length of stay was comparable between groups (median 13.27 days [IQR 10.67] vs. 13.25 days [IQR 11.55]; *p* = 0.930), as was discharge destination, with 113/201 (56.2%) sarcopenic and 110/220 (50.0%) non-sarcopenic patients discharged home (*p* = 0.202). Overall, 55/486 patients died within 30 days and 109/486 within 90 days. In unadjusted logistic regression, sarcopenia was associated with increased odds of 30-day mortality (OR 4.96, 95% CI 2.44 to 10.10; *p* < 0.001) and 90-day mortality (OR 4.01, 95% CI 2.47 to 6.52; *p* < 0.001).

**Fig. 2:**
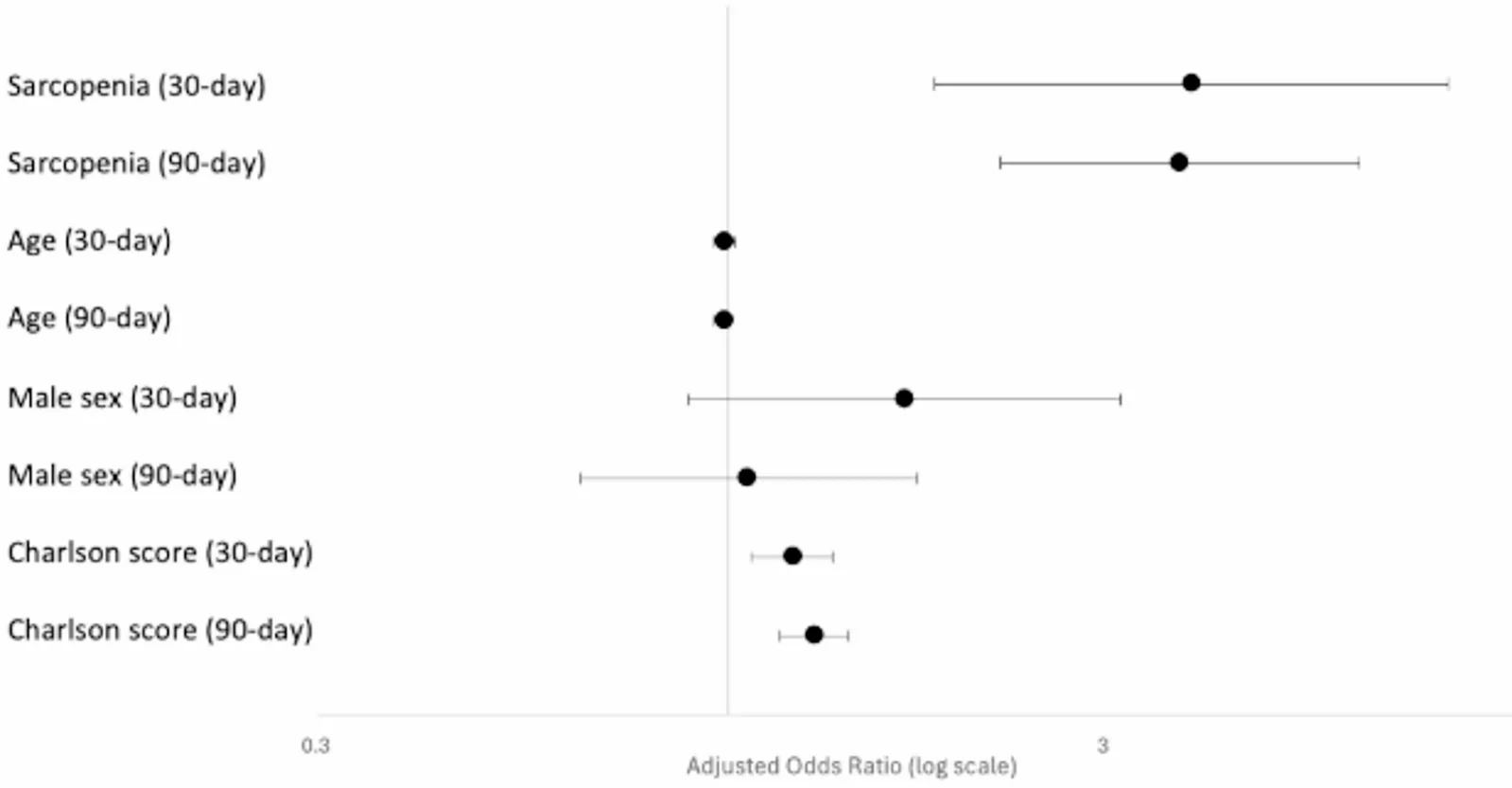
Forest plot of adjusted odds ratios and 95% confidence intervals for predictors of 30-day and 90-day mortality. The vertical line indicates OR = 1

After adjustment for age, sex and CCI, sarcopenia remained independently associated with both 30-day mortality (adjusted OR 3.88, 95% CI 1.83 to 8.22; *p* < 0.001) and 90-day mortality (adjusted OR 3.75, 95% CI 2.22 to 6.35; *p* < 0.001; Table 2; Fig. 2). CCI was also independently associated with 30-day mortality (adjusted OR 1.21 per point, 95% CI 1.07 to 1.36; *p* = 0.002) and 90-day mortality (adjusted OR 1.29 per point, 95% CI 1.16 to 1.42; *p* < 0.001), while age and sex were not independently associated with mortality in the adjusted models. Both logistic regression models demonstrated acceptable calibration by Hosmer-Lemeshow testing.

**Fig. 3:**
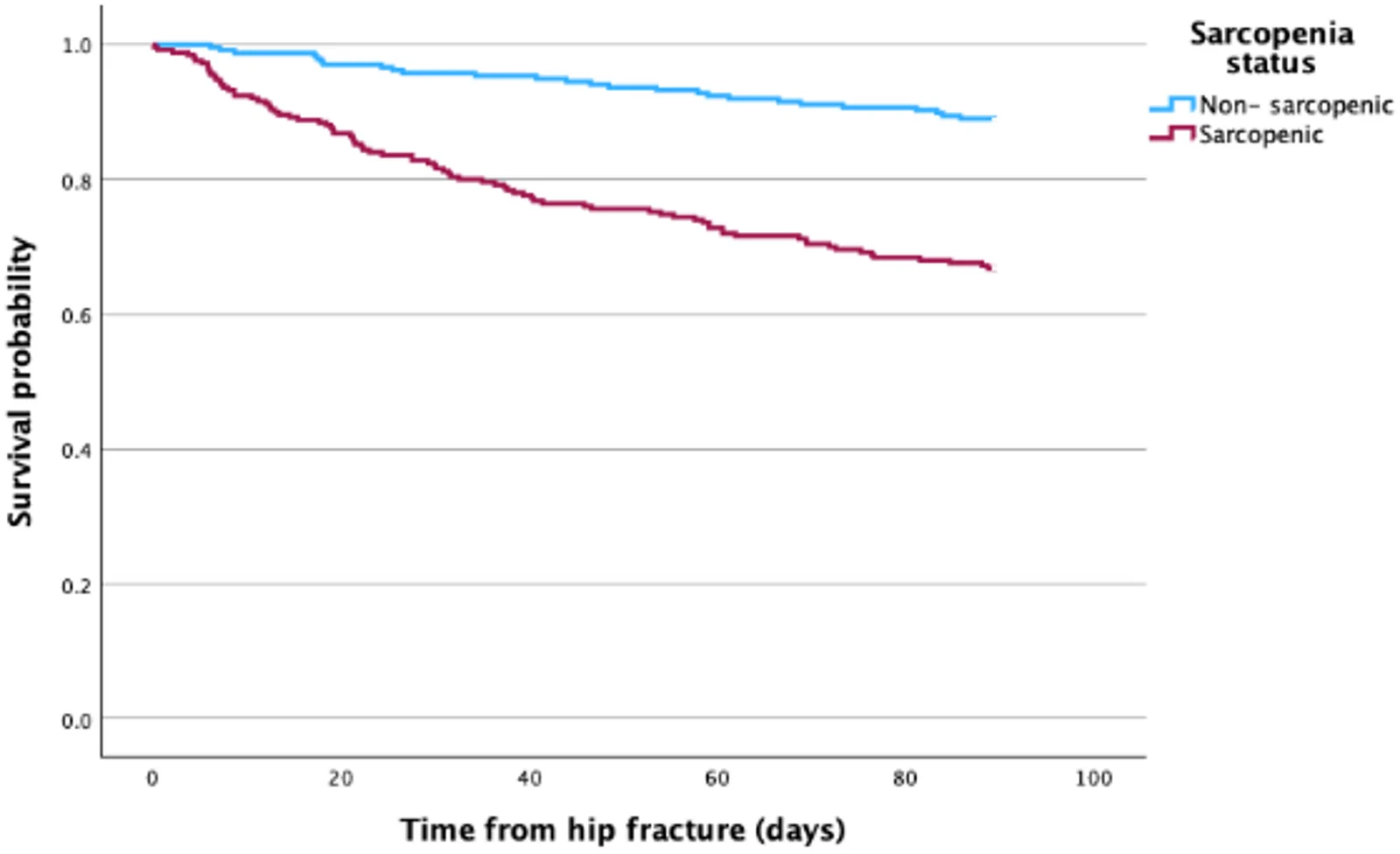
Kaplan-Meier survival curves comparing 90-day survival between sarcopenic and non-sarcopenic patients following hip fracture

Kaplan-Meier analysis demonstrated lower 90-day survival among sarcopenic patients compared with non-sarcopenic patients (log-rank χ^2^ = 35.71, df = 1; *p* < 0.001; Fig. 3). Cox regression similarly showed an increased hazard of death within 90 days in sarcopenic patients in unadjusted analysis (HR 3.52, 95% CI 2.27 to 5.48; *p* < 0.001) and after adjustment for age, sex and CCI (adjusted HR 3.19, 95% CI 2.01 to 5.06; *p* < 0.001; Table 3). CCI was also independently associated with 90-day mortality in the adjusted Cox model (HR 1.22, 95% CI 1.13 to 1.32; *p* < 0.001).

**Table 1:** Baseline demographic and clinical characteristics by sarcopenia status

Variable	Non-sarcopenic (*n* = 236)	Sarcopenic (*n* = 250)	*P*-value
Age, mean ± SD (years)	79.7 ± 8.8	81.1 ± 9.1	0.081
Sex, n (%)			< 0.001
Female	190 (80.5%)	113 (45.2%)	
Male	46 (19.5%)	137 (54.8%)	
Charlson comorbidity index score, mean ± SD	6.43 ± 2.24	6.95 ± 2.49	0.017
AMT, median (IQR)	9(4)	9 (4)	0.146
Haemoglobin, mean ± SD (g/L)	119.02 ± 19.12	117.09 ± 20.31	0.300
Malignancy, n (%)			0.176
No	139 (58.9%)	132(52.8%)	
Yes	97 (41.1%)	118(47.2%)	
Pre-admission mobility, n/N (%)			0.751
Independent/minimal aid	140/234 (59.8%)	147/247 (59.5%)	
Limited mobility	92/234 (39.3%)	96/247(38.9%)	
Non–ambulatory	2/234 (0.9%)	4/247 (1.6%)	
Pre-admission residence (care facility), n (%)	26 (11.0%)	33 (13.2%)	0.685
ASA, n/N (%)			0.211
Low anaesthetic risk	33/212 (15.6%)	25/208 (12%)	
Moderate anaesthetic risk	126/212 (59.4%)	116/208 (55.8%)	
High anaesthetic risk	53/212 (25%)	67/ 208 (32.2%)	
4AT score, median (IQR)	1 (2)	1 (2)	0.898

a. Values are presented as mean ± SD, median (IQR), or n/N (%), calculated using available data. AMT, Abbreviated mental test; 4AT, 4 A’s Test; ASA, American society of anesthesiologists physical status classification; SD, standard deviation; IQR, interquartile range

**Table 2:** Multivariable logistic regression for 30-day and 90-day mortality

Predictor	30-day aOR	95% CI	*p* value	90-day aOR	95% CI	*p* value
Sarcopenia	3.88	1.83 to 8.22	< 0.001	3.75	2.22 to 6.35	< 0.001
Age, per year	0.99	0.96 to 1.02	0.522	0.99	0.96 to 1.01	0.335
Male sex	1.68	0.89 to 3.15	0.109	1.06	0.65 to 1.74	0.816
Charlson comorbidity index score	1.21	1.07 to 1.36	0.002	1.29	1.16 to 1.42	< 0.001

a. aOR, adjusted odds ratio; CI, confidence interval. Reference groups were non-sarcopenic patients and female sex

**Table 3:** Cox proportional hazards regression for 90-day mortality

Model and variable	HR	95% CI	*p* value
*Unadjusted model*			
Sarcopenia	3.52	2.27 to 5.48	< 0.001
*Adjusted model*			
Sarcopenia	3.19	2.01 to 5.06	< 0.001
Age, per year	0.99	0.97 to 1.01	0.299
Male sex	1.07	0.71 to 1.61	0.740
Charlson comorbidity index score	1.22	1.13 to 1.32	< 0.001

a. HR, hazard ratio; CI, confidence interval. Patients alive at 90 days or who died after 90 days were censored at 90 days

## Discussion

In this retrospective cohort of patients admitted with hip fracture, CT-derived sarcopenia was independently associated with early mortality. After adjustment for age, sex and CCI, sarcopenic patients had higher odds of 30-day and 90-day mortality, with poorer 90-day survival supported by Kaplan-Meier and Cox regression analyses. This consistent association across analyses suggests that low skeletal muscle mass may capture sustained physiological vulnerability beyond immediate perioperative risk. This is biologically plausible, as sarcopenia is associated with reduced physiological reserve and impaired tolerance of acute stress following major injury [12].

Although baseline characteristics were largely comparable, sarcopenic patients had a higher CCI and a greater proportion of males. These findings are consistent with recognised associations between sarcopenia, chronic disease and male sex in CT-based studies [13–16]. CCI was also independently associated with 30-day and 90-day mortality, supporting its expected contribution to postoperative risk. Importantly, sarcopenia remained independently associated with mortality despite adjustment for CCI, suggesting that CT-defined low skeletal muscle mass provides prognostic information beyond conventional comorbidity measures.

Sarcopenia was not significantly associated with acute length of stay or discharge destination. These outcomes may be less sensitive than mortality, being influenced by rehabilitation capacity, discharge planning, social care availability and NHS pathways.

The present findings are consistent with wider evidence from opportunistic CT-derived skeletal muscle assessment in older orthopaedic and trauma populations. Park et al. reported that low L3 skeletal muscle mass was independently associated with increased 30-day mortality in elderly trauma patients [17], while Xia et al. demonstrated increased short-term and longer-term mortality among sarcopenic trauma patients, without consistent length-of-stay associations [18]. This mirrors the present study, in which sarcopenia was associated with mortality but not broader inpatient recovery measures. Evidence within hip fracture populations remains more heterogeneous: Li et al. identified CT-derived skeletal muscle index as an independent predictor of one-year postoperative mortality [19], whereas Kim et al. found no significant association with one-year mortality in a smaller cohort, although longer-term mortality was higher among sarcopenic patients [20]. Differences in cohort size, healthcare setting, imaging methodology, sarcopenia thresholds and follow-up duration may explain these discrepancies.

The supplementary baseline comparison with patients without pre-fracture CT imaging provides further context for interpretation. Patients in the included CT cohort represented a clinically distinct subgroup, with higher comorbidity burden, higher recorded malignancy prevalence and a higher-risk ASA profile despite being slightly younger. AMT scores were also slightly higher in the included CT cohort, suggesting that this group was not uniformly more impaired across all measured domains. Overall, the requirement for available pre-fracture abdominal CT imaging may have selected patients with greater underlying morbidity or different patterns of healthcare utilisation. The findings should therefore be interpreted most directly in relation to hip fracture patients with available pre-fracture abdominal CT imaging, rather than assumed to be fully generalisable to the entire screened hip fracture population.

This study has several strengths, including a relatively large cohort, balanced sarcopenic and non-sarcopenic groups, objective CT-based muscle assessment and clinically meaningful mortality endpoints. However, several limitations should be acknowledged. The retrospective single-centre design limits causal inference, and restriction to patients with available pre-fracture abdominal CT imaging introduces selection bias and may limit generalisability. CT scans obtained up to one year before fracture may not fully reflect skeletal muscle status at admission, as muscle mass may have changed between imaging and injury, introducing potential temporal misclassification. The CT-based definition used in this study does not capture the full European Working Group on Sarcopenia in Older People diagnostic framework [1], as it measures skeletal muscle mass but not muscle strength or physical performance, and height-normalised indices could not be used because height data were frequently unavailable. This limits comparability with studies using combined structural and functional definitions or height-adjusted skeletal muscle indices. Although CT measurements were performed by trained assessors following a common predefined segmentation protocol, inter-rater reliability was not formally assessed because the assessors evaluated separate scan subsets rather than an overlapping sample; consequently, measurement reproducibility could not be quantified. Clinical applicability may vary across healthcare systems depending on imaging availability, hip fracture care pathways and post-discharge support. Some covariate data were incomplete, and residual confounding cannot be excluded. Despite these limitations, the findings indicate that CT-derived low skeletal muscle mass has prognostic relevance in this selected hip fracture cohort.

## Conclusion

CT-defined sarcopenia was independently associated with increased 30-day and 90-day mortality following hip fracture, capturing vulnerability beyond chronological age and comorbidity burden. Opportunistic CT assessment may support early postoperative risk stratification where imaging is already available but should complement rather than replace functional assessment. Future studies should determine whether targeted nutritional and resistance-based rehabilitation can improve outcomes in sarcopenic hip fracture patients.

## Supplementary Information

Below is the link to the electronic supplementary material.

Supplementary Material 1

## Data Availability

The data that support the findings of this study were derived from routinely collected hospital records and the National Hip Fracture Database. Restrictions apply to the availability of these data, which were used under local institutional audit approval for the current study and are not publicly available. Individual-level data cannot be shared due to patient confidentiality and data governance requirements.

## References

[1] Cruz-Jentoft AJ, Bahat G, Bauer J, Boirie Y, Bruyère O, Cederholm T et al (2019) Sarcopenia: revised European consensus on definition and diagnosis. Age Ageing 48(1):16–31 10.1093/ageing/afy169 PMC6322506 30312372

[2] Martin FC, Ranhoff AH (2020) Frailty and sarcopenia. Orthogeriatrics: the management of older patients with fragility fractures 53–65

[3] Lang T, Streeper T, Cawthon P, Baldwin K, Taaffe DR, Harris T (2010) Sarcopenia: etiology, clinical consequences, intervention, and assessment. Osteoporos Int 21(4):543–559 10.1007/s00198-009-1059-y PMC2832869 19779761

[4] Prado CM, Purcell SA, Alish C, Pereira SL, Deutz NE, Heyland DK et al (2018) Implications of low muscle mass across the continuum of care: a narrative review. Ann Med 50(8):675–693 10.1080/07853890.2018.1511918 PMC6370503 30169116

[5] Paes VM, Ting A, Masters J, Paes MV, Graham SM, Costa ML (2025) A systematic review of evidence regarding the association between time to mobilization following hip fracture surgery and patient outcomes. Bone Jt Open 6:741–747 10.1302/2633-1462.67.BJO-2024-0243.R1 PMC12221528 40602785

[6] Yeung SS, Reijnierse EM, Pham VK, Trappenburg MC, Lim WK, Meskers CG et al (2019) Sarcopenia and its association with falls and fractures in older adults: a systematic review and meta-analysis. J cachexia sarcopenia muscle 10(3):485–500 10.1002/jcsm.12411 PMC6596401 30993881

[7] Zhang J-Z, Liu C-H, Shen Y-L, Song X-N, Tang H, Li H (2025) Sarcopenia in trauma patients: A systematic review and meta-analysis. Ageing Res Rev 104:102628 10.1016/j.arr.2024.102628 39674376

[8] He Q, Xia W (2024) Analysis of the current status of computed tomography diagnosis of sarcopenia. Archives Med Science: AMS 21(2):374 10.5114/aoms/191297 PMC12087324 40395884

[9] Shen W, Punyanitya M, Wang Z, Gallagher D, St.-Onge M-P, Albu J et al (2004) Total body skeletal muscle and adipose tissue volumes: estimation from a single abdominal cross-sectional image. J Appl Physiol 97(6):2333–2338 10.1152/japplphysiol.00744.2004 15310748

[10] Chianca V, Albano D, Messina C, Gitto S, Ruffo G, Guarino S et al (2022) Sarcopenia: imaging assessment and clinical application. Abdom Radiol 47(9):3205–3216 10.1007/s00261-021-03294-3 PMC8536908 34687326

[11] Derstine BA, Holcombe SA, Ross BE, Wang NC, Su GL, Wang SC (2018) Skeletal muscle cutoff values for sarcopenia diagnosis using T10 to L5 measurements in a healthy US population. Sci Rep 8(1):11369 10.1038/s41598-018-29825-5 PMC6063941 30054580

[12] Fuggle N, Shaw S, Dennison E, Cooper C (2017) Sarcopenia. Best Pract Res Clin Rheumatol 31(2):218–242 10.1016/j.berh.2017.11.007 PMC5768235 29224698

[13] Xia W, Luo K, Gu Z, Hu J, Liu X, Xiao Q (2024) Correlational analysis of sarcopenia and multimorbidity among older inpatients. BMC Musculoskelet Disord 25(1):309 10.1186/s12891-024-07412-2 PMC11034126 38649917

[14] Gong G, Wan W, Zhang X, Liu Y, Liu X, Yin J (2019) Correlation between the Charlson comorbidity index and skeletal muscle mass/physical performance in hospitalized older people potentially suffering from sarcopenia. BMC Geriatr 19(1):367 10.1186/s12877-019-1395-5 PMC6929451 31870318

[15] Lee JS, Kim YS, Kim EY, Jin W (2018) Prognostic significance of CT-determined sarcopenia in patients with advanced gastric cancer. PLoS ONE 13(8):e0202700 10.1371/journal.pone.0202700 PMC6101416 30125312

[16] Papadopoulou S, Tsintavis P, Potsaki G, Papandreou D (2020) Differences in the prevalence of sarcopenia in community-dwelling, nursing home and hospitalized individuals. A systematic review and meta-analysis. J Nutr health aging 24(1):83–90 10.1007/s12603-019-1267-x PMC12879112 31886813

[17] Park J, Oh Y, Kwon S, Lee J, Kim M, Choi D et al (eds) (2025) Impact of CT-defined sarcopenia on clinical outcomes in elderly trauma patients: a retrospective Korean cohort study. MDPI, Healthcare 10.3390/healthcare13182321 PMC12469919 41008450

[18] Xia W, Barazanchi AW, MacFater WS, Hill AG (2019) The impact of computed tomography-assessed sarcopenia on outcomes for trauma patients–a systematic review and meta-analysis. Injury 50(9):1565–1576 10.1016/j.injury.2019.07.001 31280971

[19] Li E-l, Hu J-s, Chen Z-h, Ma R-x, Jin C, Bu Y-t et al (2025) Based on CT scans at the 12th thoracic spine level, assessing the impact of skeletal muscle and adipose tissue index on one-year postoperative mortality in elderly hip fracture patients: a propensity score-matched multicenter retrospective study. BMC Musculoskelet Disord 26(1):21 10.1186/s12891-024-08183-6 PMC11702231 39762857

[20] Kim YK, Yi SR, Lee YH, Kwon J, Jang SI, Park SH (2018) Effect of sarcopenia on postoperative mortality in osteoporotic hip fracture patients. J Bone Metabolism 25(4):227 10.11005/jbm.2018.25.4.227 PMC6288605 30574467

