# Supplementary material for: CT-defined sarcopenia as a predictor of early mortality following hip fracture: a retrospective cohort study
Source: Eur J Orthop Surg Traumatol. 2026 Sep 30;36(1):398. doi: 10.1007/s00590-026-04981-x (PMC13627162; doi:10.1007/s00590-026-04981-x)
Supplement: Supplementary file 1 [file 590_2026_4981_MOESM1_ESM.pdf]

Supplementary Table 1. Baseline characteristics of the included CT cohort and no-CT cohort with available baseline data

Characteristic	Included CT cohort (n=486)	No-CT cohort (n=5386)	p-value
Age, median (Q1–Q3)	82 (75–87)	84 (77–89)	<0.001
Charlson Comorbidity Index, median (Q1–Q3)	7 (5–8)	5 (4–7)	<0.001
AMT score, median (Q1–Q3)	9 (6–10)	8 (4–10)	0.014
Haemoglobin, median (Q1–Q3), g/L	120 (105–132)	124 (112–135)	<0.001
**Sex, n (%)**			<0.001
Female	303 (62.3%)	3793 (70.4%)	
Male	183 (37.7%)	1593 (29.6%)	
**Recorded malignancy, n (%)**	215 (44.2%)	957 (17.8%)	<0.001
No recorded malignancy, n (%)	271 (55.8%)	4429 (82.2%)	
**ASA grade, n (%)**			<0.001
ASA 1	4 (1.0%)	91 (2.0%)	
ASA 2	54 (12.9%)	1049 (22.6%)	
ASA 3	242 (57.6%)	2598 (56.0%)	
ASA 4	116 (27.6%)	882 (19.0%)	
ASA 5	4 (1.0%)	16 (0.3%)	
ASA missing/unknown, n	66	750	

Values are presented as median (Q1–Q3) or n (%). Q1 represents the first quartile/25th percentile and Q3 represents the third quartile/75th percentile. Continuous variables were compared using the Mann–Whitney U test and categorical variables using the chi-square test. Analyses used available-case data.
